# An Equatorial Contractile Mechanism Drives Cell Elongation but not Cell Division

**DOI:** 10.1371/journal.pbio.1001781

**Published:** 2014-02-04

**Authors:** Ivonne M. Sehring, Bo Dong, Elsa Denker, Punit Bhattachan, Wei Deng, Birthe T. Mathiesen, Di Jiang

**Affiliations:** Sars International Centre for Marine Molecular Biology, University of Bergen, Bergen, Norway; University of California Berkeley, United States of America

## Abstract

Cell shape changes and proliferation are two fundamental strategies for morphogenesis in animal development. During embryogenesis of the simple chordate *Ciona intestinalis*, elongation of individual notochord cells constitutes a crucial stage of notochord growth, which contributes to the establishment of the larval body plan. The mechanism of cell elongation is elusive. Here we show that although notochord cells do not divide, they use a cytokinesis-like actomyosin mechanism to drive cell elongation. The actomyosin network forming at the equator of each notochord cell includes phosphorylated myosin regulatory light chain, α-actinin, cofilin, tropomyosin, and talin. We demonstrate that cofilin and α-actinin are two crucial components for cell elongation. Cortical flow contributes to the assembly of the actomyosin ring. Similar to cytokinetic cells, membrane blebs that cause local contractions form at the basal cortex next to the equator and participate in force generation. We present a model in which the cooperation of equatorial actomyosin ring-based constriction and bleb-associated contractions at the basal cortex promotes cell elongation. Our results demonstrate that a cytokinesis-like contractile mechanism is co-opted in a completely different developmental scenario to achieve cell shape change instead of cell division. We discuss the occurrences of actomyosin rings aside from cell division, suggesting that circumferential contraction is an evolutionally conserved mechanism to drive cell or tissue elongation.

## Abstract 2

A cytokinesis-like contractile mechanism is co-opted in a different developmental scenario to achieve cell elongation instead of cell division in *Ciona intestinalis*.

## Author Summary

The actomyosin cytoskeleton is the primary force that drives cell shape changes. These fibers are organized in elaborate structures that form sarcomeres in the muscle and the contractile ring during cytokinesis. In cytokinesis, the establishment of an equatorial actomyosin ring is preceded and regulated by many cell cycle events, and the ring itself is a complex and dynamic structure. Here we report the presence of an equatorial circumferential actomyosin structure with remarkable similarities to the cytokinetic ring formed in postmitotic notochord cells of sea squirt *Ciona intestinalis*. The notochord is a transient rod-like structure found in all embryos that belong to the phylum Chordata, and in *Ciona*, a simple chordate, it consists of only 40 cylindrical cells arranged in a single file, which elongate individually during development. Our study shows that the activity of the equatorial actomyosin ring is required for the elongation of the notochord cells. We also find that cortical flow contributes significantly to the formation of the ring at the equator. Similar to cytokinetic cells, we observe the formation of membrane blebs outside the equatorial region. Our analyses suggest that cooperation of actomyosin ring-based circumferential constriction and bleb-associated contractions drive cell elongation in *Ciona*. We conclude that cells can utilize a cytokinesis-like force generation mechanism to promote cell shape change instead of cell division.

## Introduction

Individual cell shape changes contribute significantly to morphogenesis during embryonic development [1]. The actomyosin network is a central player in the formation and transformation of functional cell shapes. Actin and myosin filaments are highly and dynamically organized in different developmental contexts, and often exist in a higher structure as a ring. The equatorial circumferential ring in cytokinesis is essential for the cell division to occur [2]. An actomyosin ring is also present in the yolk syncytial layer of zebrafish embryo, and is implicated in the epiboly movement of the enveloping cell layer [3]. In addition, the actomyosin ring is readily assembled after a wound in the cell membrane, and is responsible for the wound healing [4].

Body elongation is an essential morphogenetic process in the development of a bilateral body plan [5]. In many chordates, the elongation of an anatomically central structure, the notochord, contributes to this process [6]. In the simple chordate *Ciona intestinalis*, elongation of notochord occurs in three consecutive phases: convergent extension, individual cell elongation, and cell migration [7]–[12]. During the individual cell elongation phase, notochord cells lengthen dramatically [12],[13] and reduce their diameter substantially. A circumferential constriction forms midway in the cylindrical cell, and an actomyosin ring appears at the constriction, suggesting that the furrow results from the action of the contractile actomyosin ring, similar to the formation of the cleavage furrow in cytokinesis.

In cytokinesis, the establishment of the contractile ring is a highly coordinated process. Its initial positioning is regulated through the signaling of the anaphase spindles and the cortex [14],[15]. The actual assembly of the ring at the equator involves either localized *de novo* assembly of actomyosin filaments at the equator [16],[17] or a directional cortical flow of preexisting filaments. The cortical flow mechanism subscribes to the movement of actin and myosin filaments at the cortex, from other regions of the cell, toward the equator as a consequence of a gradient in actomyosin activity [18]–[22]. These two recruitment mechanisms are not necessarily mutually exclusive. In *Dictyostelium* cells, myosin II is recruited to the equator by both cortical flow and *de novo* association [22].

The organization of the actomyosin ring during furrow ingression is highly dynamic and constantly remodeled. Therefore, in addition to actin and myosin, the contractile ring contains other proteins that regulate actin nucleation, capping, polymerization, disassembly, cross-linking, and myosin activity [23]. The actin-depolymerizing factor (ADF)/cofilin mediates actin filament turnover [24],[25]. In *Xenopus*, cofilin is required for furrow formation [26], and in fission yeast, it is necessary for the formation and maintenance of the contractile ring [27]. Tropomyosin stabilizes actin filaments and regulates the access of other actin-binding proteins including myosins [28],[29]. α-actinin, in addition to cross-linking actin filaments, tethers actin to the membrane in the equatorial region [30],[31]. Talin, an actin-binding protein that bridges actin filaments and the adhesion apparatus [32], has also been localized to the cleavage furrow [33].

Although actomyosin is significantly enriched at the equator in dividing cells, a substantial actomyosin contractility remains outside of the furrow [34]. Formation of membrane blebs is commonly observed at the cell poles from the onset of anaphase until late cytokinesis [35]–[38]. Blebs are transient detachments of the cell membrane from the actin cortex and the streaming of cytosol that inflates the membrane [39]. The creation of a bleb requires critical levels of cytosolic pressure, membrane tension, and membrane–cortex adhesion energy [40], whereas bleb retraction requires a local contraction of the newly assembled actomyosin elements [41].

During cytokinesis, an underappreciated morphogenetic event is the cell elongation that proceeds the final cell division. This is evident, for example, in the division of the fertilized egg, where the total cell length between two poles progressively increases as the ingression deepens. In this study we examined the components and dynamics of the equatorial actomyosin network in notochord cells, and its contribution to cell elongation in the absence of cell division.

## Results

### The Notochord Elongates through the Elongation of Individual Cylindrical Cells That Are Postmitotic

**Figure 1:**
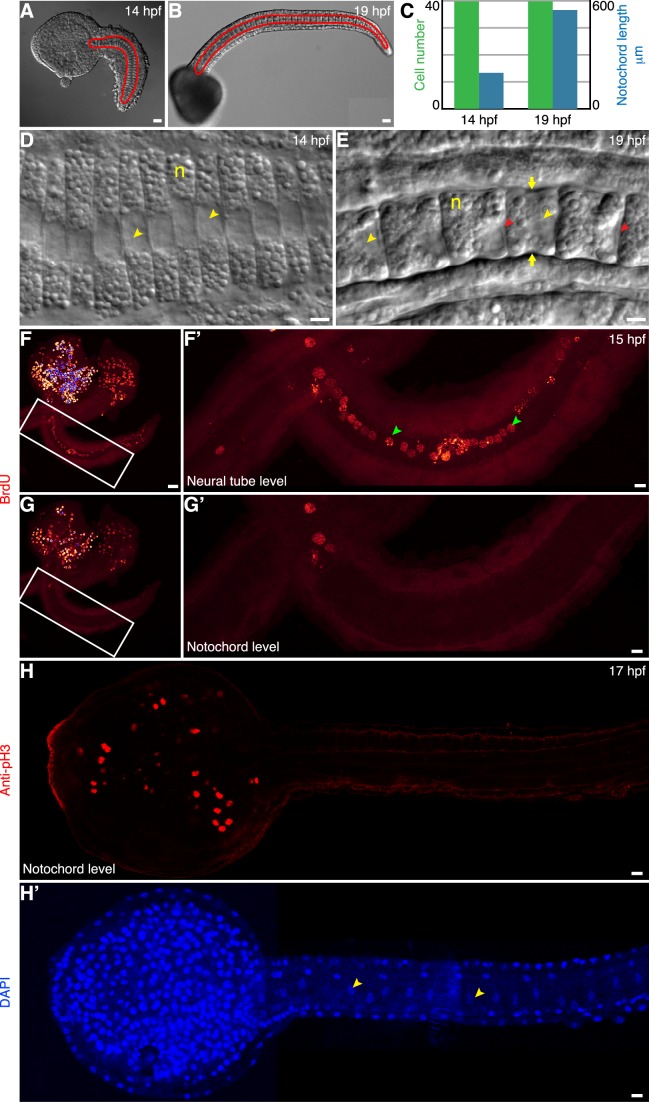
Notochord cells elongate and are postmitotic. (A) A *Ciona* embryo at 14 h postfertilization (hpf). (B) A *Ciona* embryo at 19 hpf. The notochord, outlined in red, is located centrally in the tail. (C) Between 14 and 19 hpf, the notochord cell number remains 40, whereas the notochord length increases by approximately 2.5-fold. (D) Notochord cells (n) at 14 hpf are coin shaped. (E) At 19 hpf, these cells have become drum shaped. A circumferential constriction (yellow arrow) is present at the halfway between the two ends of the cylindrical cell and persists during elongation. Yellow arrowhead indicates the nucleus, which is positioned at the posterior end of the cell. Red arrowhead indicates the lumen emerging after 18 hpf. (F, enlarged in F′) BrdU labeling of cells in the head and the tail at the level of dorsal neural tube at 15 hpf. Green arrowheads point to BrdU-positive nuclei of the cells in the neural tube. (G, enlarged in G′) A section through the notochord of the same embryo shows the absence of BrdU incorporation in the notochord cells. (H and H′) Confocal section at the level of the notochord of a 17 hpf embryo labeled with anti-pH3 and counterstained with DAPI. Anti-pH3 labels cells in the head, but not in cells of notochord, whose nuclei are visualized by DAPI (H′, indicated by yellow arrowheads). Scale bar in (D, E, F′, G′, H, H′), 5 µm; in (A, B, F, G), 20 µm. Anterior to the left.

The notochord is located in a central position in the tail of *Ciona intestinalis* embryo (Figure 1A and 1B). The specification of the notochord lineage, marked by the expression of the conserved transcription factor *brachyury*, is completed at the 110 cell stage [42],[43]. During gastrulation, 10 notochord precursors undergo two rounds of cell division and the resulting 40 cells form a monolayer epithelium anterior to the blastopore [8],[11],[44],[45]. Subsequently, in approximately 3 h up to the early tailbud stage, convergent extension takes place that rearranges the cells into a stack-of-coins configuration at 14 hpf (Figure 1A). During the next 5 h notochord cells elongate approximately 2.5-fold, whereas the cell number, the cell arrangement, and cell volume remain unchanged (Figure 1B and 1C, Figure S1A) [12]. As a result, individual notochord cells, which are overall cylindrical, change from a coin shape to a drum shape (Figure 1D and 1E). The elongation takes place along the anterior–posterior (A-P) axis of the embryo at a steady rate (Figure S1B and S1C). The nucleus of each cell, whose volume appears constant, becomes positioned against the posterior cell membrane. A circumferential constriction appears midway between the anterior and posterior ends of each cell from the onset of elongation phase, and persists throughout the elongation process. This equatorial constriction is colocalized with a circumferential band of actin and myosin filaments (Figure S2) [46], which is similar to the actomyosin ring present at the cleavage furrow of dividing cells and responsible for cytokinesis (Figure S3). In contrast to cytokinesis, however, no cell division occurs in *Ciona* notochord. Because the formation of a cleavage furrow is invariably preceded by an S phase and mitosis, we asked if cryptic cell cycle events could have taken place in notochord cells. Specifically we examined if DNA synthesis corresponding to the S phase had occurred, by monitoring bromodeoxyuridine (BrdU) incorporation. While many cells in the head and the dorsal neural tube are positive for BrdU, corresponding to continuous cell proliferation in these tissues, no BrdU is incorporated in notochord cells (Figure 1F–G′). Phosphorylation of core histone H3 (pH3) at an invariant serine residue (Ser 10) is a highly conserved histone modification and correlates specifically with chromosome condensation during the prophase of mitosis [47]. Immunohistochemistry using anti-pH3 shows nuclear staining in the mitotic cells in the head, but not in the notochord (Figure 1H and 1H′). These facts show that nondividing *Ciona* notochord cells form an equatorial constriction during elongation.

### The Architecture of the Equatorial Actomyosin Ring in Elongating Notochord Cells

**Figure 2:**
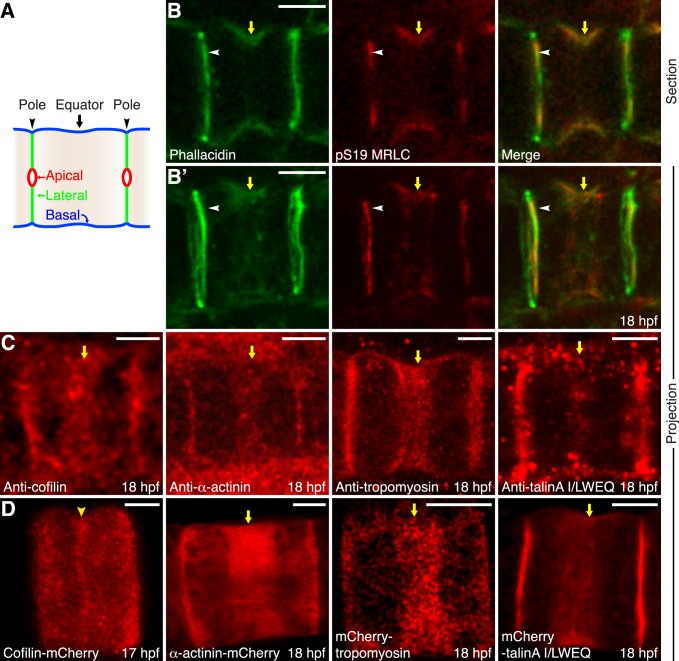
Localization of actin and actin-binding proteins in the equatorial region of notochord cells. (A) A diagram of a notochord cell at 19 hpf. The notochord cells have a cylindrical shape, with two lateral domains at the anterior and posterior ends of the cell (designated as poles, arrowheads) to form contact with two adjacent notochord cells, and a single, circumferential, basal domain that contacts the notochordal sheath. During lumen formation the center of lateral domains differentiates into apical domain that contacts emerging lumen. The morphological constriction is located at the equator of the cell (arrow). (B and B′, section and projection) Colocalization of F-actin (green) and pS19 MRLC (red) in the equatorial region of the basal domain (yellow arrows). F-actin and pS19 MRLC are also localized in the lateral domains, where they partially overlap (arrowheads). (C) Maximal projection of notochord cells shows the localization of cofilin, α-actinin, tropomyosin, and talinA revealed by immunohistochemistry. Yellow arrows indicate the accumulation of these proteins in the equatorial region. (D) Maximal projection of notochord cells shows the localization of cofilin-mCherry, α-actinin-mCherry, mCherry-tropomyosin, and mCherry-talinA in live embryos. Whereas mCherry-α-actinin, mCherry-tropomyosin, and mCherry-talinA I/LWEQ localize in a broad region (yellow arrows), cofilin-mCherry consistently localizes in a narrow line at the equator (yellow arrowhead). Anterior to the left. Scale bars, 5 µm.

The actomyosin ring is positioned in the basal cortex at a position that is equal distance from the two ends of the cell, the lateral domains (the center of which differentiates into apical domain during lumen formation) (Figure 2A). Myosin II is essential for the contractility of the actomyosin ring in cytokinesis [48]. Its motor function is activated by the reversible phosphorylation of myosin regulatory light chain (MRLC) at Serine 19 [49]. Specific antibodies against pS19 MRLC stain the cortical equatorial region of notochord cells, where they colocalize with phallacidin-labeled actin filaments (arrows in Figure 2B and 2B′). Both components are also present and partially overlap at the lateral domains (arrowhead in Figure 2B). We previously employed microarray analysis to profile notochord cell gene expression at the mid-tailbud stage and identified multiple actin binding proteins that are either specifically expressed or highly enriched in the notochord (unpublished data) [50]. Among them are *Ciona* homologs of cofilin, α-actinin, tropomyosin, and talin. A combination of immunohistochemistry and fluorescent fusion proteins reveal that these proteins are present at the equatorial cortex of *Ciona* notochord cells (Figure 2C and 2D). Noticeably, whereas α-actinin, tropomyosin, and talin fluorescent fusion proteins occupy a wide equatorial region in live embryo, cofilin-mCherry is more restricted to the equator. In addition, fluorescent protein-tagged *Ciona* IQGAP, anillin, and septin 2 are also localized in the equatorial cortex of elongating cells (Figure S4). Thus, the localization of actomyosin contractile elements and regulatory proteins in the notochord equatorial region resembles remarkably the contractile ring at cleavage furrow of a dividing cell.

### Dynamic Membrane Deformations During Notochord Elongation

**Figure 3:**
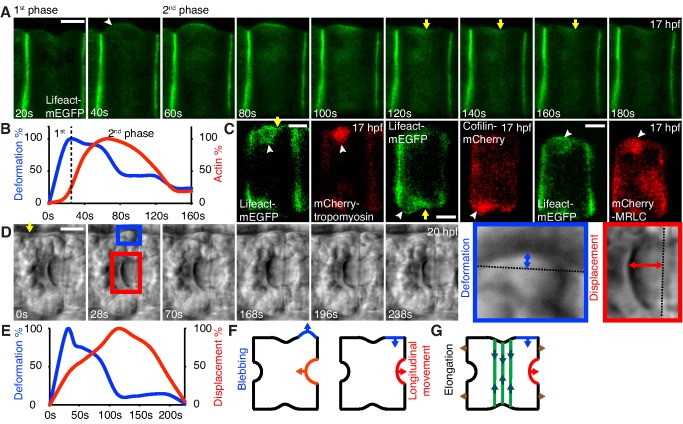
Basal local contractions in notochord cells during elongation. (A) Time-lapse frames of a lifeact-mEGFP–expressing notochord cell forming a bleb (indicated by an arrowhead) at 17 hpf (see also Movie S1). Yellow arrow indicates equatorial constriction. (B) Kinetics of the membrane deformation (in % of the maximal deformation) and relative cortical actin intensity (in % of maximal fluorescent intensity) at the bleb. The bleb goes through a fast expansion phase in approximately 27 s (*n* = 14) and a slow retraction phase in subsequent 182 s. (C) Colocalization of tropomyosin, cofilin, and MRLC with lifeact-mEGFP at a bleb (arrowheads). (D) Time-lapse Nomarski images of a bleb (enlarged in blue box), and the movement of apical/luminal membrane (enlarged in red box) in a notochord cell (see also Movie S2). (E) The kinetics of basal membrane deformation (blue double-headed arrow in D, expressed as % of the maximal deformation) and luminal membrane displacement (red doubled-headed arrow in D, between apex of luminal domain and artificial line running through the lateral domain, expressed as % of maximal displacement). Temporal cross-correlation analysis of the membrane movements shows a high correlation between temporal profiles of basal blebbing and apical membrane displacement (mean <R> = 0.76±0.05, *n* = 4), indicating that they have similar overall dynamics. The bleb retraction precedes the outbound movement of apical membrane by 49.87±23.92 s. (F) A model in which blebbing at the basal circumference causes the apical/luminal membrane to displace along the longitudinal axis. (G) A model in which the local contractile forces from bleb retraction at the basal domain are partially registered by equatorial contractile ring, which serves as a ratchet and contributes to cell elongation. Scale bars, 5 µm.

During cell elongation, we observed frequent membrane deformations at the basal surface. Time-lapse movies of notochord cells expressing lifeact-mEGFP revealed two phases of membrane deformation, a fast inflation phase, which lasts 26.71±9.80 s (*n* = 14), followed by a slow retraction phase, which is completed in 181.50±34.29 s (Movie S1, Figure 3A and 3B). The cortex of the membrane is devoid of actin during its inflation, similar to blebs observed in other systems, suggesting a detachment of the membrane from the actin cortex due to a transient increase of intracellular pressure [41]. Actin is recruited to the bleb cortex after the membrane deformation has reached its maximum and begins its slow recovery (Figure 3A and 3B). It is known that the retraction of a bleb involves the reconstitution of the actin cortex and the recruitment of myosin to power an active contraction event [39],[41]. We asked if bleb retraction can constitute a contraction in notochord cells at the basal surface by examining the molecular components of a bleb during its recovery. Indeed, tropomyosin, cofilin, and MRLC are recruited to the bleb very early during the recovery of the membrane deformation (Figure 3C), before actin appears in the bleb. These results suggest that the retraction of bleb-like membrane deformation in notochord cells represents individual contractions at the basal surface.

### Individual Local Basal Contraction Correlates with the Cell Shape Change Along the A-P Axis

Basal bleb-like contractions occur throughout the elongation phase, at very similar frequencies (0.42±0.05/cell/min, mean ± s.e.m., *n* = 42) regardless the developmental times and regions of the notochord (Figure S5). Most blebs form beside the equatorial constriction. The average size of a deformation is 37.39±3.3 µm^2^ (*n* = 17), covering only a small area (5%) of the basal membrane (734.81±45.88 µm^2^, *n* = 6), indicating that the contractile activity associated with bleb retraction is local, and applies a discrete compression inward. To test if these basal contractions (transverse loading) can be converted into forces pushing along the longitudinal axis and thus potentiating cell elongation (axial strain), we examined the behavior of the ends of notochord cells when a basal bleb occurs. At the early stage of lumen formation, both equatorial constriction and actomyosin ring persist, and notochord cells continue to elongate (Figure S1 and Figure S5) [46]. Extracellular luminal pockets emerge between adjacent notochord cells and are enclosed by newly differentiated apical/luminal domains. Time-lapse movies show a displacement of the apical membrane following the retraction of a basal bleb (Movie S2, Figure 3D). As the basal bleb forms, the apical membrane moves toward the center of the bleb-bearing cell along the longitudinal axis, then slowly returns to its previous position after the bleb recovers (Figure 3E). To relate these two events we conducted temporal cross-correlation analysis of the membrane movements. Correlation between temporal profiles of basal blebbing and apical membrane displacement is high (mean <R> = 0.76±0.05, *n* = 4), indicative of similar overall dynamics. Moreover, the bleb retraction precedes the outbound movement of apical membrane by 49.87±23.92 s. This result suggests that a discrete local contraction during bleb retraction at the basal surface can be converted to a longitudinal pushing force that produces strain along the direction of cell elongation (Figure 3F).

### Formation of the Actomyosin Ring through Cortical Flow

**Figure 4:**
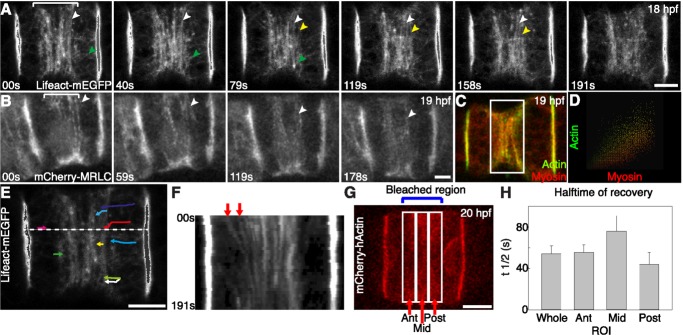
Cortical flow contributes to the formation of the equatorial actomyosin ring. (A) Time-lapse projections of a notochord cell showing that lifeact-mEGFP–labeled actin filaments (white and yellow arrowheads follow two circumferential actin filaments) move toward and align parallel to the equator in the equatorial region (bracket) (see also Movie S3). In addition to the circumferential long actin filaments, between the lateral domain and the equatorial region, a population of longitudinally oriented short filaments (green arrowhead follows a single filament) move into the equatorial region, where they join the circumferential filaments. (B) Dynamic movement of mCherry-MRLC–labeled myosin filaments toward the equator in the equatorial region (bracket). White arrowhead follows a myosin filament. (C) Confocal section (1 µm) of a notochord cell double labeled for actin (lifeact-mEGFP) and myosin (mCherry-MRLC) shows that actin filaments and myosin filaments are present simultaneously in the contractile ring. (D) Colocalization analysis of the equatorial region (box in C) shows that a significant amount of actin filaments and myosin filaments do not colocalize. (E) Manual tracking of single actin filament movement (color-coded) in a notochord cell (see Movie S3). The average velocity is 33.9±4.9 nm/s (mean ± s.e.m., *n* = 9). (F) Kymograph of actin filament movement (indicated by arrows) based on Movie S3 at the location indicated by the dash line in (E). The velocity is 29.5±10.5 nm/s, *n* = 2. (G and H) Dynamics of actin filament movement revealed by FRAP. (G) A projection of a mCherry-hActin–expressing cell that was photobleached. The photobleach mark was made over the entire equatorial region, and the halftime for recovery was determined in both the whole region, and separately in the anterior (ant), middle (mid), and posterior (post) regions, and is shown in (H). Recovery is faster in the anterior and posterior regions than in the middle region. This confirms that actin filaments flow toward the equator. Actin movement velocity is 40.8±3.8 nm/s, *n* = 4, calculated from the recovery kinetics of the whole region. Scale bars, 5 µm.

To address which mechanism underlies the formation of equatorial contractile rings in notochord cells, we analyzed the dynamics of actin and myosin filaments in live embryos. Actin behavior was followed with several probes: human actin N-terminally tagged to mCherry (mCherry-hActin), lifeact-mEGFP, and mCherry-utrophin. The latter two consist of small peptides derived from the actin binding protein Abp140 of *S. cerevisiae* and human utrophin, respectively, and bind to endogenous actin without interfering with its dynamics [51],[52]. To visualize myosin, we expressed mCherry-MRLC. These tagged proteins have the same localization patterns as endogenous proteins and serve as reliable probes for endogenous structures (compare Figure 2B and Figure 4, Figure S6). To determine if cortical flow is involved in the recruitment of actin to the equatorial plane, we collected time-lapse movies of elongating notochord cell expressing lifeact-mEGFP (Figure 4A). In order to avoid cytoplasmic signal and to record only the mobile elements at the basal cortex, five Z-sections (0.5 µm/section) from the basal surface were taken and projected. Lifeact-mEGFP reveals a highly dynamic flow of circumferential actin filaments, which emerge at the boundaries of the equatorial region, toward the equator (Figure 4A, white and yellow arrowheads follow specific filaments, and Movie S3). In addition, these films demonstrate the presence of short actin filaments that emerge from the lateral domains. These filaments are initially oriented along the longitudinal axis of the cell, and travel toward the equator. As they approach the equatorial region, they reorient, align, and merge with the circumferential filaments (green arrow follows one short filament in Figure 4A). A similar flow of circumferential actin filaments was observed using mCherry-utrophin (Figure S6 and Movie S4). The 3D time-lapse recordings of notochord cells expressing mCherry-MRLC show that the cortical flow is not only restricted to actin. Myosin form circumferential filaments and move in a similar fashion toward the equator (Figure 4B and Movie S5). Although actin and myosin colocalize in filament bundles in the equatorial region (Figure 4C), intensity correlation analysis indicates that they are also separately present (Figure 4D, ROI shown in Figure 4C). Several methods were used to further determine the dynamics of actin filaments. We manually tracked single actin filaments in the time-lapse projection movie of a cell expressing lifeact-mEGFP (Figure 4E). The average velocity is 33.9±4.9 nm/s (*n* = 9, Table S1). A kymograph generated from the movie confirmed the highly dynamic and polarized cortical flow of circumferentially oriented actin filaments within the equatorial region (Figure 4F), with a calculated velocity of 29.5±10.5 nm/s (*n* = 2, Table S1). Next, we measured the dynamic turnover of actin by fluorescence recovery after photobleaching (FRAP) in notochord cells expressing mCherry-hActin. As shown in Figure 4G, we photobleached a region that contained the whole equatorial circumferential actin filaments (blue bracket). The total fluorescence prior to bleach fully recovers, with a halftime of recovery of 54.24±7.59 s (*n* = 4, Figure 4H). To ascertain whether movement of actin toward the equator contributes to fluorescence recovery, we determined recovery halftimes separately for peripheral and central regions. Recovery is faster in the anterior and posterior regions than in the middle region. The average velocity of the filaments based on FRAP is 40.8±3.8 nm/s (*n* = 4, Table S1). These results together indicate that cortical flow contributes considerably to the formation of the actomyosin contractile ring of notochord cells. The movement of actin and myosin filaments is continuous without oscillation, and the constriction shows no relaxation. The flow persists for at least 5 h as cells elongate; however, we did not observe any accumulation of actomyosin filaments at the equator. Phallacidin staining of actin filaments at different time points confirms that the width and the intensity of the actin ring remains relatively constant (Figure S2).

### Actin Disassembly by Cofilin Is Required for Cell Elongation

**Figure 5:**
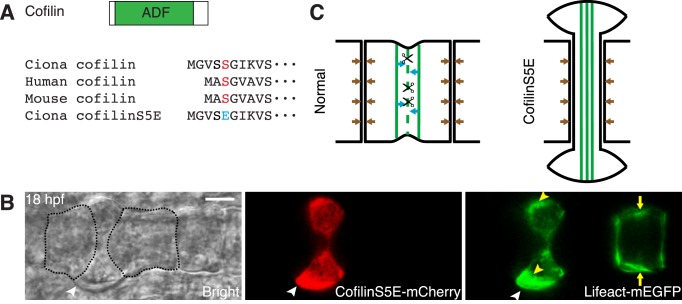
Role of cofilin in notochord cell elongation. (A) Structure of *Ciona* cofilin (top) and design of cofilinS5E mutant (bottom). ADF, actin-depolymerizing factor domain. (B) Phenotype of cofilinS5E-mCherry–expressing notochord cell (median section) at 18 hpf. Two cells flanking the cofilinS5E-mCherry–expressing cell, which is indicated by a white arrowhead, are outlined by dashed lines. They are able to elongate, and exert pushing forces along the longitudinal axis, squeezing the cofilinS5E cell to assume a dumbbell shape. Actin accumulates abnormally off the equator in the cofilinS5E cell (yellow arrowheads), instead of at the equatorial region seen in a cell not expressing the mutant (yellow arrows). (C) A model suggesting that cofilinS5E disrupts the turnover of actin filaments at the equator and the flux of actin network, resulting in an elongation failure. Scissors indicate the removal, by cofilin, of actin filaments (green lines), which move toward the equator. Brown arrows indicate the longitudinal pushing forces exerted by each notochord cell at its neighbors. Scale bars, 5 µm.

Previous work has shown that notochord in embryos treated with latrunculin B or blebbistatin fail to elongate [46]. The ubiquitous presence of actomyosin in the embryo does not allow us to exclude that the notochord phenotype is secondary to a defect elsewhere. We therefore took a genetic approach and disrupted components of actomyosin network specifically in the notochord using a notochord-specific promoter. The lack of an accumulation of actin filaments at the equator during 5 h of elongation, despite the continuous flow, suggests that an actin turnover mechanism operates in the notochord cells, similarly to cytokinesis. This is consistent with the restricted localization of cofilin, a known actin severing factor, at the equator (Figure 2C and 2D). The activity of cofilin is regulated by its phosphorylation state. Phosphorylation of an amino-terminal serine (Ser 3 in human nonmuscle cofilin) prevents cofilin from binding to actin and thus impairs its function [53],[54]. P-cofilin can be mimicked by mutating Ser 3 into glutamate. The phosphomimetic mutant S3E has been shown to act as a dominant negative [55] that inhibits the dephosphorylation of endogenous cofilin and ADF, presumably through sequestering cofilin-specific phosphatase [56],[57]. Most multicellular organisms have multiple cofilin/ADF genes; *Ciona*, however, has only one, which has a homologous serine at position 5 (Figure 5A). We therefore generated a S5E mutant, and expressed it under the control of multidom or brachyury promoter [58] via electroporation into one-cell stage embryos, which results in mosaic expression in the notochord cells. Mutant protein was not observed until 14 hpf (Figure S7D); therefore, the expression of the dominant negative does not affect the division of notochord precursors cells (Figure S7E). At 18 hpf, 37% of notochord cells (*n* = 97) expressing cofilinS5E have abnormal morphology (Figure 5B and Figure S7B, S7C), whereas cells expressing wild-type cofilin are normal (*n* = 39) (Figure S7A). Abnormal cells appear either shortened with a larger diameter, dumbbell shaped, or excluded from the notochord. We also co-electroporated lifeact-mEGFP under the brachyury promoter, which allows us to monitor the actin localization in both wild-type and mutant cells in the same embryo (Figure 5B). Two wild-type cells flanking the cofilinS5E-expressing cell are able to elongate, and exert pushing forces along the longitudinal axis, squeezing the cofilinS5E cell to assume a dumbbell shape. Actin accumulate abnormally off the equator in the cofilinS5E cell (yellow arrowheads in Figure 5B), in contrast to the usual localization at the equatorial region, as seen in a wild-type cell (yellow arrows in Figure 5B). We interpret these phenotypes as results of the disruption of actin filament disassembly and of the actin flux within notochord cells. As a consequence of this disruption, there is less equatorial constriction and less overall pressure in the cell. When the neighboring wild-type cells continuously exert longitudinal pressure along the A-P axis, the mutant cell ultimately becomes squeezed (Figure 5C).

### The Lack of Cell Elongation Is Correlated with the Absence of an Equatorial Contractile Ring

To address the role of the contractile ring at the equator in cell elongation, we examined the cells at the ends of the notochord. The first cell (cell 1) and last cell (cell 40) of the notochord are unique because they have only one notochord cell as neighbor. This asymmetry determines that the end cells in the subsequent lumen formation phase form one apical/luminal domain at the side of notochord cell/cell contact, in contrast to the other 38 cells, which create two apical/luminal domains at the opposite ends of the cells (Figure 2A) [12],[59]. We examined morphology and actin filament dynamics by collecting time-lapse movies of the ends of notochord expressing lifeact-mEGFP (Figure S8A, Movie S6). Cells anterior to cell 40 possess an equatorial actin ring, form circumferential constriction, and elongate, whereas cell 40 accumulates actin filaments at its posterior tip. No circumferential constriction is present in cell 40, and it does not elongate. Intriguingly, the time-lapse movie shows dynamic unidirectional movement of actin filaments toward the posterior tip of this cell, in contrast to the bidirectional movement of actin toward the equator in anterior cells. The lack of elongation was observed at both ends of the notochord (Figure S8B, S8C, S8E). The different behavior exhibited in the end cells is unlikely the result of differential cell sizes because the notochord cell division is equal and the placement of cells is locally random [11],[13],[44], but is caused by the absence of an equatorial contractile mechanism. To confirm this, we artificially generated end cells by cutting the embryo at random positions before the elongation phase. The newly created end cells invariably failed to form a circumferential constriction and to elongate (Figure S8D). These results together support an essential role for the equatorial actomyosin ring in notochord cell elongation.

### α-actinin Is Required for Normal Cell Elongation

**Figure 6:**
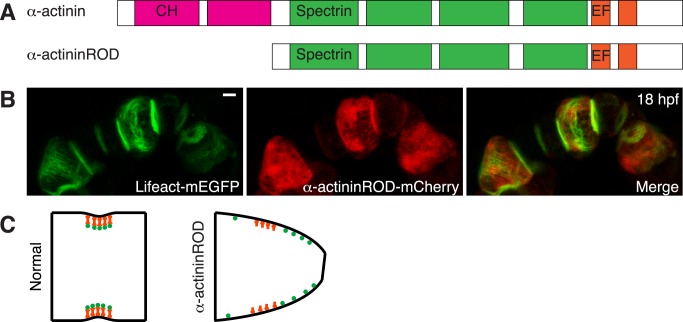
Role of α-actinin in notochord cell elongation. (A) Structure of *Ciona* α-actinin and design of the α-actininROD mutant. *Ciona* α-actinin has two calponin homology domains (CH) at the carboxyl terminus, four spectrin-repeats (Spectrin) in the central region, and calmodulin-like (CaM) domain with two EF-hand motifs at the amino terminus. α-actininROD mutant lacks CH domains that have been shown to mediate actin binding. (B) Phenotype of α-actininROD–expressing notochord cells (maximal projection) at 18 hpf. Cells expressing the α-actininROD mutant have a wedged shape. Circumferential actin filaments are not restricted to the equatorial region. (C) A model of α-actinin (red) regulating the placement of actin filaments (green, showing cross-section) and cell elongation. In normal cells, α-actinin tethers actin filaments to the equatorial membrane through its actin- and membrane-binding activity. α-actininROD mutant fails to associate with actin filaments but nevertheless can occupy the equatorial membrane tether sites, therefore disrupting the anchoring of the actin filaments in the equatorial region, consequently resulting in mislocalized contraction. Scale bar, 5 µm.

α-actinin has been implicated in cytokinesis in different organisms. It localizes to the cleavage furrow in both fungi and animal cells [31],[60]–[64], and regulates actin dynamics and controls the rate of furrow progression [31],[64]–[68]. α-actinin, through the interaction of spectrin domains, forms antiparallel rod-shaped dimers, with two actin-binding heads at the opposite ends of the complex [30]. The spectrin repeats in the rod domain also serve as binding platform for a variety of transmembrane proteins [69]. The *Ciona* α-actinin has the typical domain organization of vertebrate α-actinin (Figure 6A). We generated a truncated α-actininROD mutant by removing the N-terminal head (Figure 6A). α-actininROD lacks the ability to bind actin, but has been shown to form heterodimers with endogenous α-actinin. The mutant is dominant-negative and causes the displacement of endogenous α-actinin and cytokinesis defects [65]. Expressed under the brachyury promoter, the mutant does not affect the cell division of notochord precursors and notochord convergent extension (Figure S9D). However, of cells expressing α-actininROD (*n* = 145), 33% are impaired (Figures 6B and S9B–D) at the elongation stage. At early phases, actin filament movement is highly dynamic but disorganized (Movie S7), with an increase of bleb movement. At later phases, mutant cells do not elongate normally, but become asymmetric, with the constriction shifted toward one end of the cells (Figure 6B). The circumferential actin filament bundles move along the A-P axis over the entire basal surface (Movie S8). None of these phenotypes occur in cells expressing wild-type α-actinin (*n* = 33, Figure S9A).

### Conservation of the Equatorial Circumferential Actin Ring

**Figure 7:**
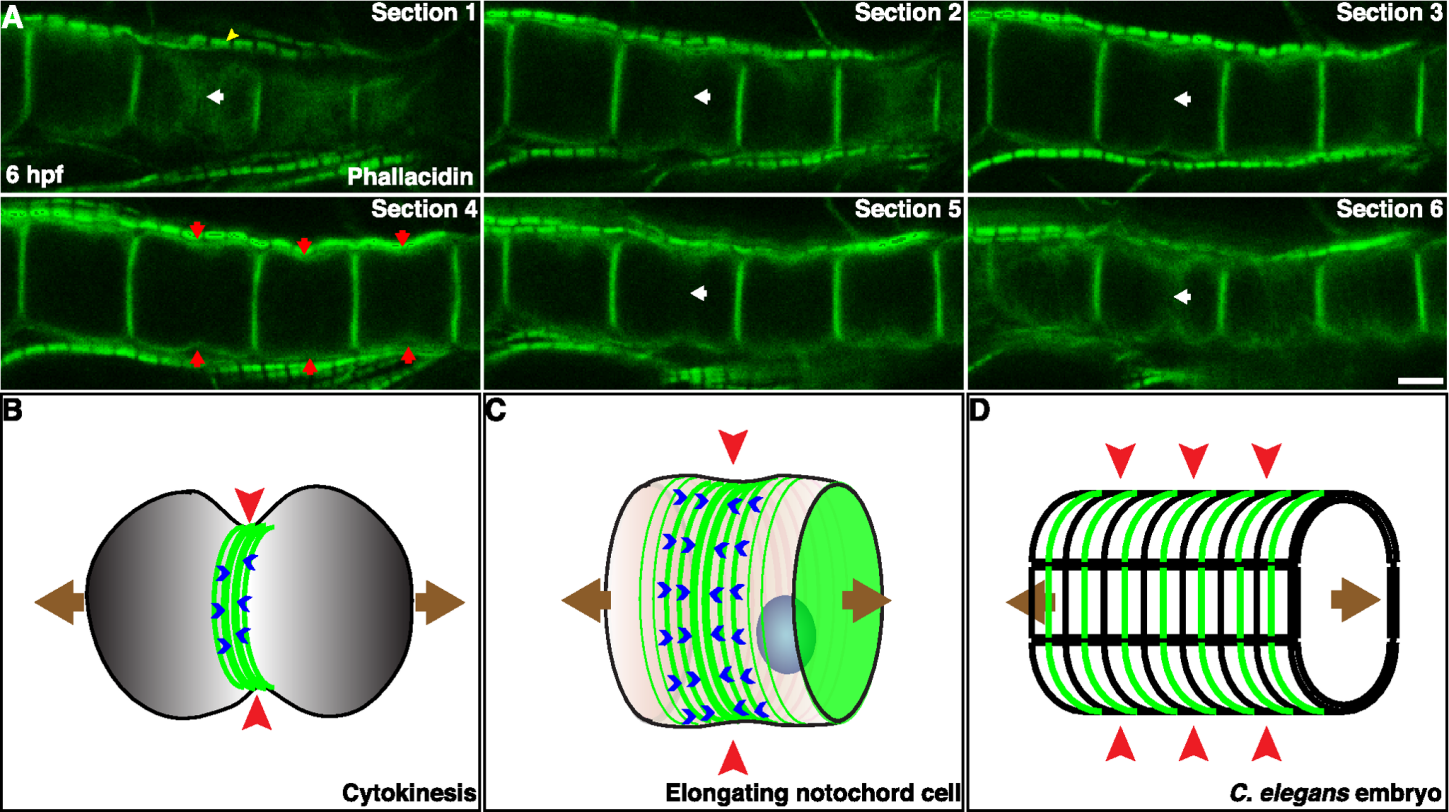
Conservation of equatorial actin ring and circumferential contraction as a common and mechanistically scalable biophysical solution for anisotropic shape change. (A) Confocal sections of elongating notochord cells in an appendicularian *Oikopleura dioica* embryo stained with phallacidin show the presence of an equatorial constriction (red arrows) and actin ring (white arrows). Yellow arrowhead indicates the actin filaments in muscle sarcomeres. (B) The equatorial circumferential constriction (red arrowhead) created by an actomyosin ring (green lines) in a dividing cell causes cell elongation (brown arrows) before cell division. Blue arrowheads indicate filament flow. (C) In notochord cells, an actomyosin ring (green lines) causes an equatorial constriction (red arrowhead), which contributes to the cell elongation (brown arrows). (D) During *C. elegans* embryogenesis, numerous circumferential actin filaments (green lines) are present at the outer surface of the hypodermal cells. The elongation of the embryo (brown arrows) is accomplished by the contractility of these actin filaments squeezing the embryo circumferentially (red arrowheads) at the scale of the entire embryo. Scale bar, 5 µm.

To explore if equatorial constriction by circumferential actomyosin network activity is a general mechanism from an evolutionary perspective, we examined the organization of actin filaments in notochord cells in *Oikopleura dioica*, an appendicularian separated from the ascidian lineage possibly during the Cambrian evolution [70]. The genomic architecture of *Oikopleura* has drastically diverged and highly modified since the last common ancestor [71]. The *Oikopleura* notochord has only 20 cells after embryonic development, and despite of having a similar morphology to ascidians, utilizes remarkably different genetic tool kits for its development [72]. Nevertheless, a circumferential basal constriction is present in the equatorial region of elongating notochord cells (red arrows in Figure 7A), where an actin ring also localizes (white arrows in Figure 7A).

## Discussion

Our results demonstrate that the *Ciona* notochord actomyosin ring possesses a well-organized molecular architecture that is strikingly similar to that of the cytokinetic contractile machinery. Dynamic cortical flow contributes to the formation of the ring. Actin-based bleb-like membrane deformations occur at the basal surface. The elongation of *Ciona* notochord cells arises from a concerted effort of two force-generating mechanisms: equatorial constriction and repeated local basal contractions. Thus, the postmitotic notochord cells exploit significant components of the complex cytokinetic machinery not for cell division but for cell elongation.

This mechanism permits the growth in length of tissues in which constituent cells are arranged in single file, a common arrangement in development. The cell division machinery being co-opted in *Ciona* notochord is likely to be more extensive, and not exclusively for cell elongation, but also for the subsequent establishment of bipolarity with two apical domains and two extracellular lumens at the opposite ends of the cylindrical cells, and for the bidirectional migrational movement that connects the lumens [12],[59]. It is interesting to note signs that some cell cycle events may also take place in notochord cells. These include the expression of the *Ciona* homolog of the replication factor CDC45 (Figure S10A) [73], and duplication and bipolar deposition of centrosomes (Figure S10B), visualized by the localization of EB1 [74],[75], despite the absence of other essential regulators and an S phase. Thus, many cell-cycle–related mechanisms may have evolved independent functions that are still unappreciated.

### Similarities and Differences between Elongating Notochord Cells and Cytokinesis

In animal cells, contractile elements are supplied in part by cortical flow from other regions of the cell to the equator [18],[19],[21],[22],[76],[77]. We show that cortical flow of actin filaments also contributes considerably to the formation of the actomyosin contractile ring in elongating notochord cells (Figure 4). The concentration of activated myosin in the equatorial region (Figure 2B) and the reorientation of short actin filaments (Figure 4A) lend support to the model that cortical flow is driven by a contractility gradient, and heightened contractility can orient the filaments to form a ring [78],[79]. The velocity of filament movement lies in the same range (tens nm per second, Table S1) as the speed observed during cytokinesis [20],[35]. Myosin filaments feature a similar directed movement. Several studies suggest that the recruitment of actin and myosin to the equator in dividing cells is at least partly independent [48]. Similarly, circumferential actin and myosin filaments only partially colocalize in notochord cells, indicating that myosin filaments do not simply slide along actin filaments toward the equator.

It has been shown that cofilin plays an important role in actin turnover during cytokinesis [25],[80],[81]. The failed furrow formation and cell elongation in notochord cells expressing cofilin mutant indicate that cofilin performs a similar function in regulating the dynamics of the contractile ring. In contrast to other actin binding proteins that are distributed over a wide equatorial region, cofilin is enriched in a narrow band precisely at the equator. Cortical actin filaments flowing in from both sides may encounter cofilin last, and be disassembled in this narrow region. α-actinin is crucial for the regulation of furrow progression during cytokinesis [31]. Due to its ability to cross-link actin filaments and to bind membrane proteins [30],[69], it is also involved in the positioning of the ring during cytokinesis in fission yeast [63], and expression of α-actininROD in mammalian cells causes a displacement of the actin ring and accelerated cytokinesis [65]. Similarly, in notochord cells expressing the α-actininROD mutant, equatorial localization of the contractile ring is disturbed, and the circumferential actin filaments roam over the entire basal surface (Movie S8). Overexpression of ROD mutant may saturate the membrane actin filament tethering sites, and disrupt the anchoring of contractile actin filaments to membrane at the equator, resulting in a shift of constriction away from the equator (Figure 6C). The presence of tropomyosin and talin in the equatorial region of notochord cells adds further to the resemblances between the notochord contractile ring and the cytokinetic contractile ring. Tropomyosin may regulate the stability of the actin filaments [28],[82], thereby confining the actin disassembly by cofilin in a narrow zone at the equator [83],[84]. Talin may connect the actomyosin filaments with the plasma membrane in notochord cells, as it does during cytokinesis [85].

Cytokinesis is invariably preceded by an interphase and mitosis, in which the duplication and separation of chromosomes occur, respectively. In contrast, notochord cells establish a functional equatorial contractile ring without an S phase and mitosis, suggesting that significant aspects of cytokinesis can be controlled independently from events of the cell cycle. For example, the microtubule organization in elongating notochord cells bears little resemblance to that of a dividing cell. There is no central spindle, nor astral microtubules. Instead, microtubule filaments are found mainly in the membrane cortex, and a significant amount of microtubules are arranged circumferentially in the basal domain (Figure S11A) [46], similar to what is found in plants [86]. Detailed analyses reveal that circumferential microtubules are located in the lower cortex, beneath the cortical circumferential actin filaments (Figure S11C). Whereas actin filaments are highly mobile, microtubule filaments exhibit little mobility in the same time scale (unpublished data). Furthermore, disruption of microtubules with 40 µM nocodazol [46] affects neither the positioning of the equatorial actomyosin filaments (Figure S11D) nor notochord cell elongation (Figure S11E), in contrast to the treatment with myosin inhibitor blebbistatin [46], which significantly reduces cell elongation. Thus, both the establishment of the equatorial actomyosin ring and cell elongation are independent from the microtubule network. In addition, the nucleus is invariably positioned at the posterior end of each cell (except cell 40). Hence, the establishment of an equatorial actomyosin ring can only be regulated through a spindle-independent mechanism. Such a mechanism has been alluded by the observation of polar lobe formation in molluscan early embryogenesis. For example, *Ilyanassa obsoleta* embryos at first cleavage form two contractile rings. In addition to the cleavage furrow in the animal hemisphere between the spindle poles, a second constriction forms in the vegetal hemisphere below the spindles and orthogonal to the first furrow, creating a transient polar lobe that represents an elongation of the cell [87]–[89]. In cytokinesis, the mitotic apparatus has been shown to be dispensable because the disruption or removal of the mitotic apparatus at anaphase or later does not affect cell cleavage [2],[90]. A spindle-independent pathway has recently been identified, which utilizes cortical polarity signals to position the ring and furrow, and partly redundantly regulates asymmetric cell division of the *Drosophila* neuroblasts with the central spindles [91]. In addition, polar cortical contractility and blebs can modulate the position of the ring in dividing cells in culture [92]. These and our observation indicate that the capacity and potential of the basic cellular and molecular toolkit used to position the ring is still underappreciated.

### A Novel Mechanism for Cell Elongation

Several observations indicate that the equatorial actin ring in notochord cells is contractile. The actin filaments are associated with phosphorylated myosin filaments. Inhibition of myosin activity abrogates the furrow formation. Disruption of the ring dynamics by the cofilin mutant results in a failure to form the furrow. Mislocalization of the ring by α-actinin mutant causes the furrow to shift to one side. Furthermore, the flow of actin filaments toward the equator is consistent with the idea that the contractility of actomyosin ring at the equator powers the flow [78]. The circumferential actomyosin ring in cytokinesis is essential for furrow formation and ingression, which in most cases is associated with an elongation of the cell before the final division (Figure 7B). Four pieces of evidence support that the contractile ring at the equator contributes to the notochord cell elongation. First, the notochord in embryos treated with latrunculin B or blebbistatin fails to elongate [46]. Second, cells expressing the cofilin dominant negative, which interferes with the actin dynamics, fail to elongate, and are pressed by neighboring cells with normal contractile ring activity. Third, the end cells, which normally do not assemble a ring at the equator, do not elongate. Lastly, in cells expressing α-actinin mutant, the actin filaments remain contractile but are mislocalized, and the elongation is partial and abnormal. We therefore suggest that the equatorial constriction force is transduced hydraulically to a longitudinal pushing force that drives cells to elongate within the confine of the notochordal sheath (Figure 7C). In addition, the hydraulic pressure created by the equatorial ring is likely the cause for tearing of the basal membrane off the actin cortex to form blebs, which may act as valves releasing the cortical contractility, as it occurs in cytokinesis [92].

The traditional view envisions that the actomyosin ring generates force through myosin-dependent actin filament sliding, as it occurs in muscle sarcomeres [93]. This mechanism has been complemented recently by the finding that myosin II plays an important role in cross-linking actin filaments and exerting tension [94]. Significantly, we have observed numerous local contractions on the circumferential basal surface mostly between the equatorial region and the lateral domains. This suggests that the equatorial region is under higher tension, which makes it less conducive for membrane deformation. Furthermore and intriguingly, we observed a correlation between the basal blebbing and membrane movement along the A-P axis. This temporal coupling supports that a discrete local contraction at the basal circumference can also be converted to a longitudinal pushing force that exerts at the two poles of the cell (Figure 3F). Our time-lapse movies show a return of the apical domain to its original position (Figure 3E). Cell elongation may partly result from repeated stresses originated at the basal surface over a long period, after the notochord cells, considered here as fully elastic, reach their yield point [95]. Alternatively, based on a more realistic model of the living cell as being viscoelastic [96] or poroelastic [97], frequent stresses (min) can produce sustained deformation over a large time scale (hours) [98],[99]. The contractile ring, in addition to providing a direct role in driving cell elongation, may act as a tension-based barrier to channel force (or displacement of cytoplasm) to the cell's poles, and provide a ratchet mechanism to maintain a fraction of elongation gained through each bleb retraction event (Figure 3G). The biophysical mechanism for the cell elongation is likely to be more complex. For example, it has been demonstrated that actomyosin flow generates reverse directional forces that drive the spreading of the enveloping cell layer over the yolk cell in zebrafish gastrulation [3]. This suggests that the equator-bound actomyosin flow seen in notochord cells may also generate an A-P pulling force by flow-friction mechanism, which in turns participates in notochord cell elongation.

### Circumferential Contraction as a Biophysical Solution for Symmetric Anisotropic Shape Change Is a Widespread Phenomenon that Appeared Many Times in Evolution and Is Mechanistically Scalable

Appendicularians, together with ascidians, belong to the Tunicata subphylum, which diverged from the vertebrates very early during chordate evolution. The third Chordate subphylum, Cephalochordata, diverged before the split of tunicates and vertebrates [100]. Intriguingly, notochord cells in the amphioxus *Branchiostoma lanceolatum* also contain transverse actin and myosin filaments that are contractile [101],[102], although amphioxus notochord cells do not elongate, and the actin filament contractility facilitates animal locomotion. It thus appears that the notochord of early chordates is a contractile device. The notochord in ascidians and appendicularians, which has a small number of cells, achieves tissue elongation by circumferential contraction, whereas in amphioxus it elongates by cell proliferation and applies the contractility of the actomyosin elements for a different purpose.

A noncytokinetic circumferential actin ring has also been found in other tissue types—for example, during polar lobe formation, and in the myoid segment of retina rods in certain fish species, where it is essential for the dramatic elongation of the rod after light stimulation [103],[104]. In addition, early workers have noted that a number of isolated individual amphibian cells, particularly the neural plate cells, can elongate, and intriguingly, they show local constriction rings traveling the length of the cell as successive waves [105]. Similar travelling constriction rings that contain actomyosin filaments have also been observed in leucocytes during cell shape changes and migration [106]–[109]. Furthermore, circumferential actin filaments are found in multiple cell types in plant roots, and are important for the anisotropic growth of individual cells and the extension of the root [110]–[112]. At a tissue level, numerous circumferential actin filaments are present at the outer surface of the hypodermal cells in the *C. elegans* embryo during development (Figure 7D). The elongation of the body is accomplished by the contractility of these actin filaments squeezing the embryo circumferentially [113]. Finally, the collective effect of basal contraction of individual follicle cells, through the action of a transverse array of actomyosin filaments along the circumference, drives the elongation of the *Drosophila* ovary [114]. Taken together, we propose that the circumferential contraction as a biophysical solution for anisotropic shape change, specifically bipolar extension of a symmetric biological unit, is a widespread phenomenon that appeared many times in evolution and is mechanistically scalable, as it can operate in single cells as well as in a whole embryo.

## Materials and Methods

### Animals and Fertilization

*Ciona intestinalis* were collected from several fjords around Bergen, Norway, or purchased from Roscoff Marine Station, France. Animals were maintained in running filtered seawater. Gametes from several individuals were surgically removed and mixed. After fertilization, the embryos were dechorionated with 1% sodium thioglycolate and 0.05% protease E as previously described [115], then washed four times in UV-treated seawater. The embryos were cultured at 16°C. *Oikopleura dioica* were obtained from cultures of the Sars Centre Appendicularia Facility. For *in vitro* fertilization, females were collected in glass dishes and left to spawn. Sperm from several males was used for fertilization. Embryos were left to develop at 19°C.

### Constructs

Cofilin, cofilinS5E, α-actinin, α-actininROD, EB1, tropomyosin, IQGAP, anillin, and septin 2 were amplified from *Ciona* cDNA using primers listed in Table S2. PCR products were used to create entry clones by recombination using the pCR8/GW/TOPO system (Invitrogen). The entry clones were used to generate notochord expression constructs using destination vectors *Minos*-B3-eBra-bpFOG-B5::R1-*ccd*B/CmR-R2-mCherry [12] (for cofilin-mCherry, cofilinS5E-mCherry, α-actinin-mCherry, α-actininROD-mCherry, EB1-mCherry, IQGAP-mCherry, anillin-mCherry, and septin 2-mCherry) and *Minos*-B3-eBra-bpFOG-B5::Kozak-mCherry-R1-*ccd*B/CmR-R2 [46] (for mCherry-tropomyosin) with the Gateway cloning method (Invitrogen). The mCherry-talinA I/LWEQ, mCherry-hActin, mCherry-UtrCH, lifeact-mEGFP, and mCherry-MRLC, ensconsin-3XGFP expression clones were described previously [46]. We also created a cofilinS5E-mCherry expression construct by recombining the entry clone into *Minos*-B3-multidom-B5::R1-*ccd*B/CmR-R2-mCherry that was modified from *Minos*-B3-eBra-bpFOG-B5::R1-*ccd*B/CmR-R2-mCherry, in which the promoter eBra-bpFOG was replaced with the notochord-specific promoter from *Ciona* multidom gene [58].

### DNA Electroporation

Electroporation was performed as described previously [42] with some modifications. Plasmid DNA (80 µg in 80 µl) was mixed with 400 µl 0.95 M mannitol, then added to 300 µl dechorionated fertilized eggs and electroporated in a 4 mm cuvette using a Gene Pulser Xcell System (BIO-RAD), with a square pulse protocol (50 V and 15 ms per pulse). After electroporation, embryos were cultured at 16°C.

### Antibodies, Immunohistochemistry, Phallacidin Staining, and BrdU Staining

Full-length cofilin and tropomyosin cDNAs from the entry clones were subcloned into pT7MAT provided by Proteogenix. Recombinant proteins were produced in *E. coli* and used to immunize mice. Antisera were affinity-purified according to the manufacturer's protocol (Proteogenix). Polyclonal rabbit anti-talinA I/LWEQ antibody was a gift from R.O. McCann. Polyclonal rabbit anti-MRLC phosphorylated at S19 (pS19 MRLC) antibody was purchased from Cell Signaling Technology (3671). Monoclonal rat anti–α-actinin antibody was obtained from Abcam (MAC276). Alexa 568-conjugated anti-rat, anti-rabbit, and anti-mouse secondary antibodies were from Invitrogen (A11077, A11011, and A11004, respectively).

For α-actinin, talin, cofilin, and tropomyosin antibody staining, *Ciona* embryos were fixed with 4% formaldehyde in filtered seawater for 3 h at 4°C, washed with PBS containing 0.1% Triton X-100 (PBST), and incubated in blocking buffer (PBST+10% goat serum) for 3 h at room temperature (RT). Embryos were incubated overnight with the primary antibodies diluted 1/200 in blocking buffer at 4°C, followed by three washes within 8 h with PBST at 4°C. Secondary antibodies, diluted 1/300, were incubated overnight at RT, followed by three washes with PBST at 4°C within 8 h. pS19 MRLC antibody staining was performed according to a published protocol [116]. After the procedure, the embryos were counterstained with 3 unit/ml BODIPY FL phallacidin (Invitrogen, B607) for 1 h at RT and washed three times.

To detect mitotic nuclei in *Ciona* embryos, we used an anti-phospho-histone H3 antibody (Ser 10, Upstate/Millipore, 06-570) at a 1/200 dilution, followed by an anti-rabbit Alexa-568-coupled secondary antibody (Invitrogen A11011, dilution 1/1,000). The embryos were counterstained with DAPI (1 µg/ml).To stain actin filaments in *Oikopleura dioica* embryos, embryos were fixed in 4% formaldehyde containing 0.5 M NaCl and 0.1 M MOPS (pH 7.5) overnight at 4°C. After several washes in PBS, samples were incubated in 2 unit/ml BODIPY FL phallacidin (Invitrogen, B607) for 7 d at 4°C.

To detect nuclei in S phase, we used the thymidine analog 5-bromo-2′-deoxyuridine-5′-monophosphate (BrdU). Live embryos at late tailbud 3–4 stages [45] were incubated for 45 min at room temperature in 5 mM BrdU in filtered sea water (FSW); then quickly rinsed and thoroughly washed in FSW twice for 10 min. Embryos were then fixed overnight at 4°C in 4% paraformaldehyde in PBS, pH 7.5 containing 0.5 M NaCl and 0.1% Tween 20 (Tw). After several washes, embryos were permeabilized with washes in PBS 0.5% Triton X100 and a 10′ incubation in acetone at −20°C. Before immunohistochemistry, samples were incubated in 4 N HCl for 10 min to denature the DNA, then washed in PBS 0.1% Tw. Embryos were blocked for 2 h at room temperature in PBS containing 0.2% Triton X100, 0.3% BSA, and 10% goat serum. BrdU incorporation was then detected using a primary mouse anti-BrdU antibody (Roche, 11 170 376 001, 1/50) and secondary anti-mouse Alexa 568–coupled antibody (Invitrogen, A-11004, 1/1,000).

### Microscopy

Nomarski images were taken using a Nikon Eclipse (E800) microscope equipped with a 40× objective (NA 1.00) and a SPOT RtKE CCD camera (Diagnostic Instruments), and a Zeiss Imager.M2 microscope equipped with a 100× objective (NA 1.30) and a pco.sensicam camera (Pco Imaging). Confocal images were taken using a Leica TCS SP5 confocal laser-scanning microscope equipped with 40× oil-immersion and 63× water-immersion objectives (NA 1.25 and 1.40, respectively). If necessary, embryos were sedated using 0.2% MS222 (Sigma, A5040). To visualize the dynamics of F-actin (mCherry-UtrCH and lifeact-mEGFP) and myosin filaments (mCherry-MRLC), we collected z-stacks of notochord cells at regular intervals, as specified in the movie legends. ImageJ and Leica TCS SP5 LAS AF software package were used to perform maximum projections and to construct movies. Fluorescence intensity was measured with ImageJ. The kymograph was compiled using the kymograph plugin in ImageJ. The velocity of single actin filaments was determined by calculation from the kymograph and by manual tracking in time-lapse movies using the Manual Tracking plugin in ImageJ. Colocalization of actin and myosin was analyzed using the Intensity Correlation Analysis plugin in ImageJ.

### FRAP

The Leica TCS SP5 was utilized to bleach the whole equatorial domain of notochord cells. Maximum laser power at 561 nm was used for an empirically determined number of iterations to achieve bleaching throughout the full thickness of the cortical equatorial actin signals. After bleaching, images were taken at regular intervals (between 4.5 and 9 s) with the same laser at 30% laser intensity. To calculate the halftime of recovery, the signal intensity in the region of interest (ROI) was measured over time. The background obtained from areas outside the embryo was subtracted. Bleaching during image acquisition was corrected by calculating the loss of signals in unbleached notochord cells and from the signal intensities in the image series before bleaching. The intensity was plotted as a function of time, and the half-time of recovery, *t_1/2_*, was extracted. The velocity was calculated from *t_1/2_* of the whole bleached region and the width of ¼ of the bleached region under the assumption of a directed flow of the filaments from the lateral domains toward the equator.

### Cross-Correlation Analysis

Time delays between basal blebbing and luminal membrane movement were determined by cross-correlation. We measured the relative locations of the membrane at the basal bleb and the apical/luminal domain over time using ImageJ. Resulting curves were smoothed using a bisquare algorithm implemented in SigmaPlot software (Systat Software). Cross-correlation analysis was performed applying Igor Pro (Wavemetrics) cross-correlation function.

### *In Situ* Hybridization

*In situ* hybridization was carried out as described by Wada et al. [117]. Cdc45 was amplified with 5′-ATGTTAATTACCGACCCAGTAAAGG-3′ and 5′-TTATGACATTATAGTGATGAGAGCG-3′ and cloned into pCRII TOPO (Invitrogen). The plasmid was linearized using Xho I and transcribed using DIG RNA Labeling Kit Sp6 (Roche).

## Supporting Information

Figure S1 **The rate of notochord cell elongation.** (A) Notochord cell morphology at different developmental times. Arrows indicate the equatorial constriction in the notochord cells (n). (B) Notochord cells elongate steadily over time. Length of 10 cells in the primary lineage was measured every 3 min from the completion of convergent extension to the lumen stage. The mean (± s.e.m.) is plotted against time. (C) The rate of cell elongation. Change in cell length in every 3 min is plotted against time. Regression of the data reveals a very slight decay over time, however this decrease is not statistically significant.
(EPS)

Figure S2 **Actin ring at the equator of notochord cells.** (A–D) Phallacidin staining of *Ciona* embryos from 14 hpf to 18.5 hpf. White arrows indicate the actin ring. Scale bars, 5 µm.
(EPS)

Figure S3 **Formation of actomyosin ring during cell division in** ***Ciona intestinalis*** **.** Embryos undergoing second cleavage (A) and third cleavage (B) were stained with phallacidin for F-actin (green) and anti-pS19 MRLC antibody (red). F-actin and myosin are enriched at the cleavage furrow (arrow). Progenies of each cell division are given. In (B) the embryo is viewed from the animal pole; the cleavage plane of all four cell divisions is parallel to the plane of photograph. (C–E) Cleavage furrows of dividing notochord (C and D) and muscle (E) precursors contain F-actin and pS19 MRLC (indicated by arrow) in an early gastrula stage embryo. The embryo was triple-stained with phallacidin (green), anti-pS19 MRLC antibody (red), and DAPI (blue). Ant, anterior; post, posterior; yellow star, blastopore; yellow arrowhead, mitotic DNA. (A and B), projection; (C and D), section; (E), 3D reconstruction of confocal Z-stack. Scale bars, 10 µm.
(TIF)

Figure S4 **Localization of mCherry-tagged** ***Ciona*** **IQGAP, Anillin, and Septin 2 in the equatorial region of notochord cells.** IQGAP has been shown to be expressed in *Ciona* notochord [118]. Microarray analysis of notochord cell gene expression at the mid-tailbud stage reveals the presence of anillin and septin 2, 7, and 11 in the notochord (unpublished data) [50]. (A–C), maximal projection; (A′–C′), confocal section close to the basal cell surface; (A″–C″), median section. All three proteins are localized at the equatorial constriction (yellow arrows). Anillin-mCherry is also strongly localized in the nucleus (yellow arrowhead). Scale bar, 5 µm.
(EPS)

Figure S5 **Rate of basal blebbing.** Basal membrane movement is monitored by confocal imaging of cells labeled with lifeact-mEGFP that delineates the membrane contour and actin-rich cortex. (A) Dynamics of basal blebbing. Deformation of basal membrane in five cells over 12 min at 18 hpf is plotted. (B–E) Representative plot of basal membrane deformations in a notochord cell in the primary lineage at 15 hpf (B), in the secondary lineage at 18 hpf (C), and in the primary linage at 18 hpf (D) and 19 hpf (E), over a 12-min period. The primary lineage contributes to the anterior 32 notochord cells; the secondary lineage contributes to the posterior 8 notochord cells, which are consistently smaller. (F) Rate of basal membrane blebbing at different developmental time points and in primary and secondary lineages. Primary lineage at 15 hpf, 0.46±0.03 bleb/min, *n* = 16; secondary lineage at 18 hpf, 0.36±0.04 bleb/min, *n* = 8; primary lineage at 18 hpf, 0.44±0.03 bleb/min, *n* = 4; secondary lineage at 19 hpf, 0.36±0.03 bleb/min, *n* = 6; primary lineage at 19 hpf, 0.46±0.04 bleb/min, *n* = 6. Blebbing activities at different stages of cell elongation and in different regions of notochord are not significantly different.
(EPS)

Figure S6 **Cortical flow of actin filaments revealed by mCherry-UtrCH.** Time-lapse micrographs of a notochord cell at 18 hpf showing that mCherry-UtrCH–labeled actin filaments (arrowhead follows a single filament) move toward and align parallel to the equator in the equatorial region (bracket) (see also Movie S4).
(EPS)

Figure S7 **Role of cofilin in notochord cell elongation.** (A) Expression of wild-type cofilin-mCherry. (B and C) Expression of cofilinS5E-mCherry. Arrows indicate cells with abnormal phenotype. Dashed lines in (C) outline boundary of the notochord. (D) Snapshots of an embryo expressing cofilinS5E-mCherry showing the increasing amount of cofilinS5E-mCherry during notochord development (arrowhead). All timeframes were taken with the same image acquisition settings. The cofilinS5E-mCherry signal becomes detectable at 14 hpf, after the completion of convergent extension, and 5 h after the last notochord cell division. (E) Notochord precursor cells expressing cofilinS5E divide normally, resulting in 40 notochord cells (marked by white dots). The cofilinS5E-positive cells usually fail to form an equatorial constriction, and are wider and shorter than their neighbors. Scale bars in (A–C), 5 µm; in (D and E), 10 µm.
(EPS)

Figure S8 **The absence of an equatorial contractile ring is correlated with the absence of cell elongation.** (A) Maximum projection of the posterior end of a notochord expressing mCherry-UtrCH. This image is the first frame of Movie S6. The last cell, cell 40, is significantly shorter. Equatorial actin ring and circumferential constriction, which are evident in cells 35 to 39 (yellow arrow), are absent in cell 40. Instead, actin filaments accumulate at the posterior tip of the cell (yellow arrowhead). Movie S6 shows a unidirectional movement of actin filaments toward the posterior tip, unlike the bidirectional movement of actin filaments toward the equator in cells 35 to 39. (B and C) The anterior-most cell and posterior-most cell are significantly shorter. White lines indicate the cell lengths. (D) An end cell created by cutting the embryo before the elongation has begun is significantly shorter. (E) Cells at both ends of the notochord are significantly shorter and wider. For cells 1 to 3, *n* = 9; for cells 38 to 40, *n* = 23. Scale bar, 5 µm.
(EPS)

Figure S9 **Role of α-actinin in notochord cell elongation.** (A) Expression of wild-type α-actinin. (B and C) Expression of α-actininROD. Arrows indicate cells with “wedged” phenotype. (D) Notochord precursors expressing α-actininROD divide normally, resulting in 40 notochord cells (marked by white dots). Scale bars in (A–B), 5 µm; in (D), 10 µm.
(EPS)

Figure S10 **Expression of** ***Ciona Cdc45*** **and centrosome duplication in the notochord cells.** (A) Expression of *Ciona cdc45* in the notochord cells at the early mid-tailbud stage. (B) Duplication and bipolar deposition of centrosomes, labeled with EB1-mCherry, in the notochord cells at 18 hpf. Scale bar, 5 µm.
(EPS)

Figure S11 **Role of microtubules in cell elongation and the formation of circumferential actin filaments.** Projection of a notochord cell labeled with ensconsin-3XGFP (green) and mCherry-UtrCH (red) showing the arrangement of microtubules (A) and F-actin (B) into circumferential filaments in the equatorial cortex. (C) Confocal sectioning reveals that microtubules and actin filaments do not colocalize (arrows). (D) Projection of cells labeled with ensconsin-3XGFP (green) and mCherry-UtrCH (red) after 15 and 60 min treatment with 40 µM nocodazole. (E) Percentage cell length increase within 45 min during treatment with DMSO (+39%), blebbistatin (+12%) or nocodazol (+32%); *n* = 50 for each treatment. Scale bars, 10 µm.
(EPS)

Movie S1 **Blebbing and recruitment of actin at the bleb.** Consecutive z-stacks of notochord cells at 17 hpf expressing lifeact-mEGFP (20 sections, 1 µm/section) were recorded every 19.8 s. The movie of the maximum projections has a frame rate of 3/s. The movie shows the fast extension of a bleb, followed by the recruitment of actin to its cortex, and the slow bleb retraction.
(MOV)

Movie S2 **Basal membrane deformation and luminal membrane movement during blebbing.** Nomarski recordings of notochord cells every 3.5 s, revealing membrane deformation at the basal cell cortex and the movement of the luminal membrane. The movie of a single optical section has a frame rate of 6/s.
(MOV)

Movie S3 **Movement of actin filaments labeled with lifeact-mEGFP.** Confocal z-stacks (5 sections, 0.5 µm/section) at every 6.6 s were recorded from a notochord cell for 3 min. The movie of the maximum projections has a frame rate of 3/s.
(MOV)

Movie S4 **Movement of actin filaments labeled with mCherry-UtrCH.** Confocal z-stacks (14 sections, 0.5 µm/section) at every 12 s were recorded from a notochord cell for 6 min. The movie of the maximum projections has a frame rate of 10/s.
(MOV)

Movie S5 **Myosin filament dynamics.** Consecutive z-stacks (15 sections, 1 µm/section) of notochord cells expressing mCherry-MRLC were recorded every 19.8 s. The movie of the maximum projections has a frame rate of 3/s.
(MOV)

Movie S6 **Shape and actin dynamics in the last cell.** Confocal z-stacks (14 sections, 0.5 µm/section) at every 60 s were recorded from a notochord cell for 20 min. The movie of the maximum projections has a frame rate of 10/s.
(MOV)

Movie S7 **Phenotype of α-actininROD expression at 15 hpf.** Notochord cells are also labeled with lifeact-mEGFP for actin filaments. Confocal z-stacks (16 sections, 2 µm/section) at every 17.8 s were recorded from a notochord cell for 3 min. The movie of the maximum projections has a frame rate of 8.5/s.
(MOV)

Movie S8 **Phenotype of α-actininROD expression at 17 hpf.** Notochord cells are also labeled with lifeact-mEGFP for actin filaments. Confocal z-stacks (25 sections, 1 µm/section) at every 18.8 s were recorded from a notochord cell for 3.5 min. The movie of the maximum projections has a frame rate of 11/s.
(MOV)

Table S1 **Velocity of actin filaments in cortical flow.**
(DOC)

Table S2 **PCR primers.**
(DOC)

## Abbreviations

ADF: actin-depolymerizing factor
A-P axis: anterior-posterior axis
BrdU: bromodeoxyuridine
CaM: calmodulin
CH: calponin homology
FRAP: fluorescence recovery after photobleaching
FSW: filtered sea water
hpf: hours postfertilization
MRLC: myosin regulatory light chain
PBST: PBS containing 0.1% Triton X-100
pH3: Phosphorylation of core histone H3
pS19: Phosporylation of Serine 19
ROI: region of interest
RT: room temperature
