# Supplementary figures and images for: An Equatorial Contractile Mechanism Drives Cell Elongation but not Cell Division
Source: PLoS Biol. 2014 Feb 4;12(2):e1001781. doi: 10.1371/journal.pbio.1001781 (PMC3913557; doi:10.1371/journal.pbio.1001781)

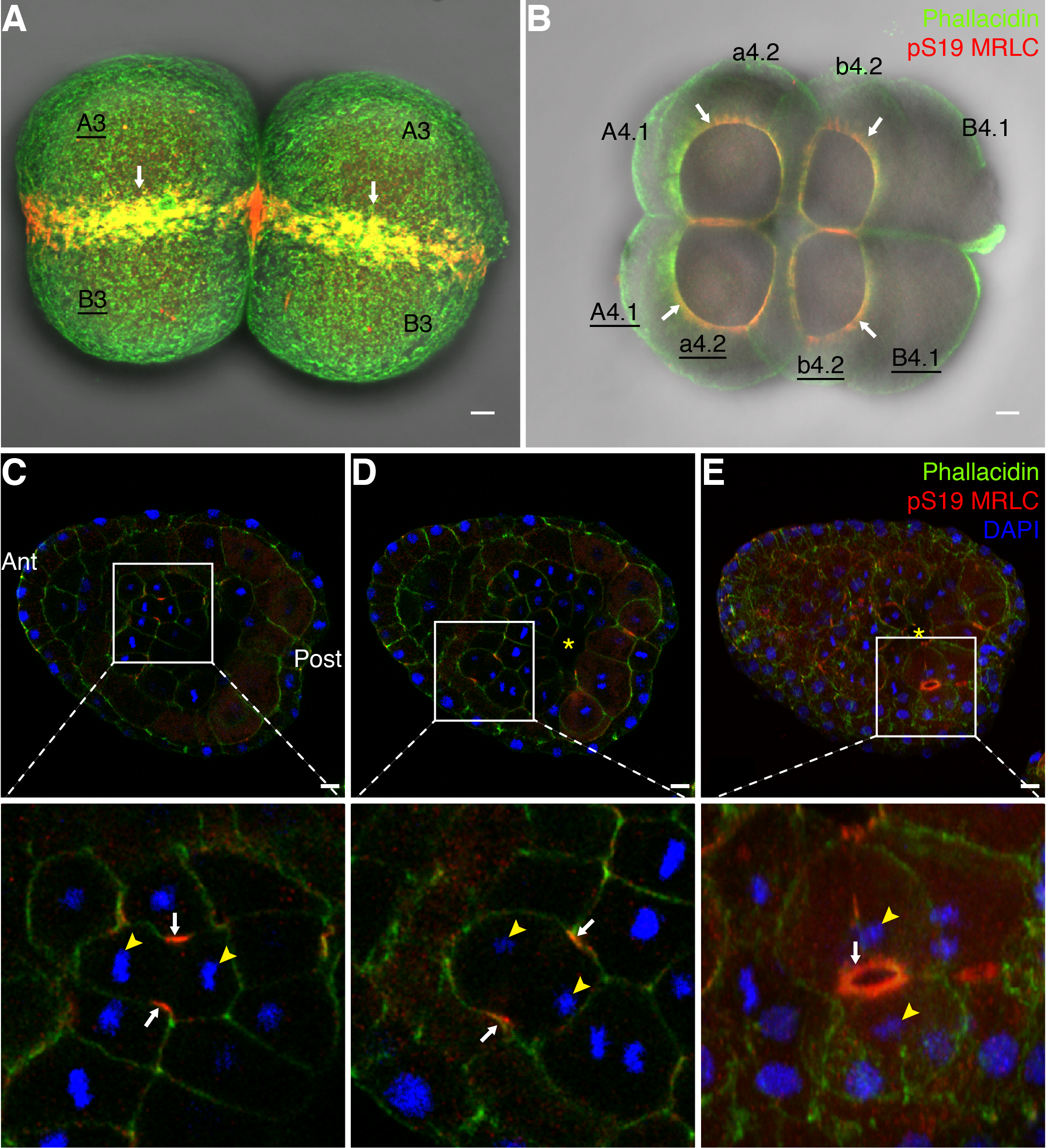

Supplement: Figure S3 — Formation of actomyosin ring during cell division in Ciona intestinalis . Embryos undergoing second cleavage (A) and third cleavage (B) were stained with phallacidin for F-actin (green) and anti-pS19 MRLC antibody (red). F-actin and myosin are enriched at the cleavage furrow (arrow). Progenies of each cell division are given. In (B) the embryo is viewed from the animal pole; the cleavage plane of all four cell divisions is parallel to the plane of photograph. (C–E) Cleavage furrows of dividing notochord (C and D) and muscle (E) precursors contain F-actin and pS19 MRLC (indicated by arrow) in an early gastrula stage embryo. The embryo was triple-stained with phallacidin (green), anti-pS19 MRLC antibody (red), and DAPI (blue). Ant, anterior; post, posterior; yellow star, blastopore; yellow arrowhead, mitotic DNA. (A and B), projection; (C and D), section; (E), 3D reconstruction of confocal Z-stack. Scale bars, 10 µm. (TIF) [file pbio.1001781.s003.tif]
