# Supplementary material for: An Equatorial Contractile Mechanism Drives Cell Elongation but not Cell Division
Source: PLoS Biol. 2014 Feb 4;12(2):e1001781. doi: 10.1371/journal.pbio.1001781 (PMC3913557; doi:10.1371/journal.pbio.1001781)
Supplement: Table S1 — Velocity of actin filaments in cortical flow. (DOC) [file pbio.1001781.s020.doc]

**Table S1.** Velocity of actin filaments in cortical flow.

| **Method** | **Sample** | **Velocity [µm/s]** | **SEM** |
| --- | --- | --- | --- |
| **FRAP** | Cell 1 | 0.051 |  |
|  | Cell 2 | 0.036 |  |
|  | Cell 3 | 0.034 |  |
|  | Cell 4 | 0.042 |  |
|  | **Mean** | **0.0408** | **3.8161e-3** |
| **Manual tracking** | Filament 1 | 0.0375 |  |
|  | Filament 2 | 0.0407 |  |
|  | Filament 3 | 0.0423 |  |
|  | Filament 4 | 0.0332 |  |
|  | Filament 5 | 0.0498 |  |
|  | Filament 6 | 0.0523 |  |
|  | Filament 7 | 0.0177 |  |
|  | Filament 8 | 0.0215 |  |
|  | Filament 9 | 0.0097 |  |
|  | **Mean** | **0.0339** | **4.8925e-3** |
| **Kymograph** | Filament 1 | 0.04 |  |
|  | Filament 2 | 0.019 |  |
|  | **Mean** | **0.0295** | **0.0105** |
|  |  |  |  |

SEM, standard error of the mean
