# Supplementary material for: An Equatorial Contractile Mechanism Drives Cell Elongation but not Cell Division
Source: PLoS Biol. 2014 Feb 4;12(2):e1001781. doi: 10.1371/journal.pbio.1001781 (PMC3913557; doi:10.1371/journal.pbio.1001781)
Supplement: Table S2 — PCR primers. (DOC) [file pbio.1001781.s021.doc]

**Table S2. PCR primers.**

| **Gene** | **Primer Pair** |
| --- | --- |
| ***Cofilin*** | 5’-ATGGGCGTAAGTTCCGGAATTA-3’  5’-TTAGACATATTCATATGCGTTACT-3’ |
| ***CofilinS5E*** | 5’-ATGGGCGTAAGTGAAGGAATTA-3’  5’-TTAGACATATTCATATGCGTTACT-3’ |
| ***α-actinin*** | 5’-CAGAAAAAATGGATGAACAGCAGTATATG-3’  5’-AAGATCAGACTCCCCATAGAG-3’ |
| ***α-actininROD*** | 5’-CAGAAAAAATGTCTGGGGCACAGAAGGCCG-3’  5’-AAGATCAGACTCCCCATAGAG-3’ |
| ***Tropomyosin*** | 5’-ATGGAAAACATTAAAATGAAAATAGC-3’  5’-TTACATTTCTCCGAGCTCGCTC-3 |
| ***EB1*** | 5’-CAGAAAAAATGGCCGCTGTGAATGTGTTT-3’  5’-ATATTCCTCCTGCTGTTCAAC-3’ |
| ***IQGAP*** | 5’-CAGAAAAAATGTCTGCTGGAGTAACCCCCGATGACACA -3’  5’-TTTTCCGAAGAATTTCTTGTTGAGGAG-3’ |
| ***Anillin*** | 5’-CAGAAAAAATGTCTGCTGGAGTAACCCCCGATGACACA -3’  5’-TTTTCCGAAGAATTTCTTGTTGAGGAG-3’ |
| ***Septin 2*** | 5’-CAGAAAAAATGCCGTCAGAGGTAGACGATTTTACACA -3’  5’-TTTAAGCGGTTTAAGTGCGTTCACATCCCA-3’ |
